# Sustaining the nursing workforce - exploring enabling and motivating factors for the retention of returning nurses: a qualitative descriptive design

**DOI:** 10.1186/s12912-024-01900-5

**Published:** 2024-04-17

**Authors:** Kumiko Yamamoto, Katsumi Nasu, Yoko Nakayoshi, Miyuki Takase

**Affiliations:** School of Nursing, Yasuda Women’s University, 6-13-1, Yasuhigashi, Asaminami-ku, 731-0153 Hiroshima, Japan

**Keywords:** Retention, Returning nurses, Enabling factors, Motivating factors, Qualitative descriptive design

## Abstract

**Background:**

The nursing shortage represents a persistent and urgent challenge within the healthcare industry. One of the most cost-effective and time-efficient solutions to address this issue is the recruitment of inactive nurses to rejoin the nursing workforce, while simultaneously ensuring the long-term sustainability of their careers following their return to work. The aim of this study is to explore the factors that facilitate the retention of nurses who have returned to work, from their perspective.

**Methods:**

To achieve this aim, a qualitative descriptive design was employed. A total of 15 registered nurses who had not practiced nursing for a minimum of three years prior to their return to work, and had been working as nurses for at least three months following their return, were selected from seven healthcare institutions using convenience sampling. Face-to-face or online semi-structured interviews were conducted, and qualitative inductive analysis was employed to analyze the collected data.

**Results:**

The analysis revealed five key themes, two of which were related to the enabling factors making it possible for the nurses to continue their work, while the remaining three pertained to the motivating factors driving the pursuit of professional careers. The two themes associated with enabling factors were identified as “Conditions and support that sustain work-life balance” and “A workplace that acknowledges my career, and encourages my growth as an experienced nurse”. The three themes related to motivating factors were entitled “Pride in reconnecting with and contributing to society,” “Cultivating confidence through incremental professional development and future envisioning,” and “Enrichment of my own and my family’s life”.

**Conclusions:**

Returning nurses constitute a valuable asset for healthcare institutions. To effectively retain these nurses, it is crucial to implement multi-dimensional approaches that enable and motivate them to sustain and enrich their professional and personal lives while continuing their work in the nursing field.

## Background

Nurses constitute a vital cornerstone of the healthcare system, assuming a foundational role in providing patient care, and notably representing over half of the entire healthcare workforce [1]. The global nurse population was estimated at 27.9 million in 2018 [1], and there was a notable growth of 4.7 million nurses between 2013 and 2018 [1]. Simultaneously, the WHO [1] reported a deficit of 5.9 million nurses in 2018, with the shortfall in the number of nurses expected to reach 10.6 million by 2030 [2]. This trend is primarily driven by the mounting demand for nursing services stemming from population aging dynamics. Moreover, the aging composition of the nursing workforce exacerbates the existing shortage of nurses. Currently, 17% of the global nursing population is aged 55 years or over [2], and projections indicate that within the upcoming decade, approximately 4.7 million nurses are expected to retire [3]. This means that an estimated annual influx of 47,000 new nurses is required just to sustain the current nursing workforce. Failure to meet this demand will probably intensify the nursing shortage at an accelerating pace. There is an immediate need for cost-effective measures aimed at mitigating the shortage of nurses.

Numerous policies have been implemented on a global scale to address the persistent shortage of nursing professionals. These policy measures encompass creating new registered nurses through education; facilitating re-entry into the nursing workforce for currently inactive registered nurses, and recruiting nurses from other countries [4]. Among the aforementioned strategies, one particularly promising approach to overcoming the nursing shortage involves the recruitment of inactive nurses, which has been implemented in many countries [4, 5]. The reintegration of inactive nurses into the labor force is advantageous in terms of cost and time, as it obviates the need to invest social capital and years of resources in educating and nurturing new nursing students. Countries have implemented Return to Practice Programs designed for inactive nurses, each varying in educational content and duration [e.g., 6, 7], and these initiatives have demonstrated notable success in augmenting the nursing labor force [8–11].

The reintegration of these nurses into the labor force holds significant importance in addressing the nursing shortage in Japan in particular. Japan is currently facing the challenge of a super-aging population, with 29.0% of its total population being 65 years and older [12]. This demographic shift has imposed increasing demands on nursing professionals, as older people often experience multiple chronic illnesses that result in physical and cognitive decline [13], necessitating substantial medical support and assistance in daily activities. In response to this demand, the Japanese government has actively pursued strategies to increase enrollment in nursing schools, reduce attrition rates, promote the retention of currently practicing nurses, and encourage inactive nurses to return to nursing practice [14]. However, the declining birth rate in Japan has led to a decrease in the number of students enrolling in nursing schools since 2018 [15]. Although the improvement in the workplace environment has contributed to a reduction in the turnover rate of full-time nursing personnel from 11.0% in 2013 to 10.6% in 2021, which is slightly lower than the average turnover rate across all occupations (i.e., 11.3%) [16], this alone cannot address the issue of the nursing shortage. Consequently, an inevitable imbalance between demand and supply persists. The Ministry of Health, Labor, and Welfare in Japan [14] projected a demand for 1.88–2.02 million nurses by 2025, when the baby boomer generation reaches 75 years old or older, while the projected supply would be 1.75–1.82 million nurses, resulting in a shortage of 60,000 to 250,000 nurses. Therefore, the recruitment of inactive nurses has emerged as a pivotal measure to rectify this imbalance promptly.

Available statistics show that there is an estimated population of approximately 700,000–860,000 inactive nurses in Japan [17], the United States [18] and Germany [19]. Several studies have demonstrated that a significant proportion of surveyed inactive nurses, ranging from 43 to 85%, expressed a desire to return to nursing practice [20, 21]. The motivations behind their return or desire to return to nursing practice encompass factors such as no longer having childcare responsibilities [22], a yearning for nursing practice [22], seeking a renewed purpose in life after completing child-rearing [23], financial incentives [10, 22, 23], and a desire to update skills and knowledge in acute care nursing [24]. Similarly, a more recent study conducted in Taiwan reported that incentives for returning to practice included the improvement of the nurse staffing level, and the provision of a safer working environment and re-entry preparation programs [20].

However, it should be noted that despite the expressed intentions, many inactive nurses have faced challenges in returning to practice as well as in sustaining their employment [25]. These challenges related to returning to work include difficulties in balancing work with childcare and household responsibilities, anxiety arising from a perceived lack of competency, concern about heavy work responsibilities, and fears of committing medical errors [15]. Consequently, previous research findings have indicated that only 57–69% of nurses who completed the Return to Practice Program were able to successfully re-enter the nursing workforce [26]. These challenges persist even after returning to work, as reported in subsequent studies [27–29], exacerbated by the absence of family-friendly working conditions, inadequate on-the-job training opportunities, and insufficient ongoing education and mental support to overcome anxiety and regain confidence [30]. As a consequence, nurses who have returned to work experience a sense of guilt toward both their colleagues and patients for perceived inadequacies in care provision, as well as feelings of guilt toward their families due to the sacrifices necessitated by their work obligations [31], all of which contribute to higher attrition rates among returners. In fact, the findings from a small-scale survey conducted in Japan revealed that 25% of nurses who participated in refresher programs and returned to work were unable to sustain their employment [32]. This retention rate is significantly higher compared to the turnover rates observed among newly graduated nurses (7.8%) and nurses with prior experience (17.7%) [16].

While it is crucial to address the barriers encountered by nurses who wish to return to practice and have successfully done so, it is equally imperative to ensure the long-term sustainability of their careers following their return to work. However, the factors that contribute to the retention of these returners have not been thoroughly investigated. For instance, Barriball et al. [33] and Elwin [27] investigated the experiences of nurses returning to practice, although their focus was primarily on the experiences within the Return to Practice Program, rather than the process of returning to the workplace itself. Conversely, Durand and Randhawa [34], Hammer and Craig [23] and Costantini, et al. [35] explored the experiences of nurses returning to work; however, they did not focus on the specific factors that facilitate retention. In fact, only a limited number of studies have endeavored to identify factors that facilitate the retention of inactive nurses. The key findings facilitating their retention were preceptors fulfilling their learning needs [28, 31], support on nursing units [31], flexible working atmosphere [28], and re-building a new family life [28] or re-negotiation with both work and home life [36]. Nevertheless, these studies are based on a relatively small sample of five to eight nurses who have returned to practice, thus leaving the possibility that some factors remain undiscovered. A comprehensive understanding of the factors that not only prompt nurses to leave their positions but also motivate them to remain is crucial for the development of strategies that ensure a sufficient nursing workforce and the provision of high-quality nursing care in countries grappling with nursing shortages.

Therefore, the aim of this study is to explore the factors that facilitate the retention of nurses who have returned to work, from their perspectives.

## Methodology

This study employed a qualitative descriptive design [37]. The qualitative descriptive approach produces “findings closer to the data as given, or data-near” [38, p. 78], without commitment to any theoretical views and without being bounded by preconceptions [38]. As such, this approach provides straightforward and comprehensive descriptive summaries of participants’ experiences and perceptions [39, 40], thus it is suitable for areas where little is known about the topic under investigation [39]. We applied this approach to investigate the factors that contributed to the retention of these returners.

### Participants

The participants were selected from seven healthcare institutions located in the southwestern region of Japan, and using convenience sampling and snowball sampling. The participants comprised re-entry nurses employed in five community hospitals and two long-term care facilities situated across metropolitan, urban, and rural areas of Japan with populations ranging from 0.4 million to 2.7 million. Inclusion criteria for the nurses were that they (1) had not practiced nursing for a minimum of three years prior to returning to work (based on the Japanese childcare policy allowing a maximum three-year leave), (2) had been working as nurses for a minimum of three months after returning to work, and (3) were able to participate in interviews conducted in Japanese. Exclusion criteria included: (1) working as nursing managers after returning to work, and (2) being without prior experience of working in Japanese healthcare institutions (i.e., those who only had overseas experience). Participants were recruited until saturation was reached, i.e., no further new information emerged during the interviews. A total of 15 participants were recruited as a result.

### Data collection

The research team approached the Directors of Nursing and obtained permission to recruit potential participants. Written statements were distributed to the potential participants to explain the purpose and methods of the study.

Semi-structured interviews (see Table 1 for the interview guide) were conducted face-to-face or online, between November 2021 and July 2022. The interview guide was developed based on the research purpose and the review of existing literature. The first author conducted all interviews because her 16-year career hiatus from nursing for child-rearing would help her establish a mutually respectful relationship with the participants and foster an environment free from intimidation. These conditions are crucial for eliciting participants’ genuine sentiments. Throughout the interviews, the author demonstrated respect and empathy toward the participants by openly sharing her own feelings. Additionally, she skilfully guided the discussions to extract the participants’ experiences, concurrently undergoing a process of reintegration in tandem with them. Conversely, the dynamic between the interviewer and participants could be impacted by the assumptions and biases inherent in the interviewer’s background. To mitigate this potential influence, data analysis was performed independently by two researchers (refer to the Data Analysis section).

The interviews were conducted in private rooms, and all sessions were audio-recorded. Nonverbal data, such as the participants’ posture during the interviews, were recorded in an observation notebook. Each participant underwent a single interview session and received a book voucher valued at ¥2500 as a token of appreciation. The interviews lasted between 18 and 49 min (Mean = 39.2 min). Audio-recorded data were transcribed verbatim.

**Table 1:** Interview guide

Opening statement	What were your concerns before returning to work?
	What made you decide to return to work?
	What kind of training did you have before returning to work?
Further exploration	What facilitated the continuation of your job?
	Were there anything good about returning to work?
	When did you feel good about returning to work?
	In what specific situations did you feel good about returning to work?
	Why did you feel good about returning to work?
	Can you tell me what kind of training you had after returning to work?
	Please tell me about the changes you have made since returning to work.
	1. about family (e.g., children, husband, parents, etc.)
	2. about yourself (e.g., lifestyle)
	3. about the job (e.g., the types of wards, job descriptions)

### Data analysis

Qualitative inductive analysis [41] was conducted. Verbatim transcripts were thoroughly reviewed to develop an overall understanding of the participants’ statements. Meaningful words and paragraphs related to the factors that had facilitated the retention of these re-entry nurses were extracted, and codes were assigned to represent the symbolic meanings of the data segments (first-cycle coding). Subsequently, the codes were compared and contrasted to group them into categories based on their similarities in meaning. These categories were further integrated into themes that captured the essence of the factors facilitating the retention of nurses who returned to the nursing workforce (second cycle coding). The first-cycle coding was conducted by the first author (KY) by utilizing her understandings of the participants’ context and their experiences. In the second cycle of coding, the first (KY)and second (KN) authors independently categorized the codes, and the congruencies or discrepancies between them were discussed among all the research team members (KY, KN, YN, and MT), who possessed nursing backgrounds and qualitative research experience. Discussion continued until consensus was reached among all the research members. NVivo12 (QSR International, Melbourne, Australia) software was used for data management.

### The trustworthiness of the study

Ensuring credibility, confirmability, transferability and dependability contributes to the trustworthiness of the study [42]. To enhance the credibility, we applied method triangulation. The interviewer (i.e., the first author) took notes on the participants’ facial expressions and eye movements during the interview, which were included in the analysis along with the verbatim transcripts of the interview data. During the analysis process, the first author repeatedly read the transcripts and observational notes to code the data. For confirmability, two researchers independently categorized the codes, and discussions among the research team took place repeatedly to ensure the elimination of any preconceptions or biases. Any disagreements that arose during this process were resolved through discussions among the research team. To enhance the transferability of the findings, participants were recruited from diverse practice areas and various regions. Furthermore, detailed information was provided regarding the participants’ characteristics and their practicing contexts. In addition, the dependability of the findings was assured by providing detailed descriptions of the data collection and analysis process.

### Ethical considerations

This study was approved by the Review Board of Yasuda Women’s University (approval number: 210007), and ethical approval was waived by the participating institutions. This study was conducted in accordance with the Declaration of Helsinki. The participants were fully informed about the study’s purpose, methods, potential risks, and benefits of participation as well as their right to decline participation or withdraw from the study. Written informed consent was obtained from each participant before the data collection. The interview schedule and location were prioritized according to the preferences of the participants, as many were balancing work and childcare responsibilities. Participants were assured that they could refrain from answering any questions that made them feel uncomfortable. Additionally, they were informed that they could end the interview session at any time if they experienced emotional distress. The collected data were securely stored in a locked cabinet, and pseudonyms were used to maintain the participants’ anonymity and protect their privacy.

### Findings

All 15 eligible participants were female. The reasons cited for leaving employment were childbirth/child-rearing in 11 cases, caring for older family members in three cases, and pursuing a postgraduate degree in one case. The range of length of clinical experience before leaving employment was 3–20 (Mean = 8.2, SD = 4.2) years, that of career breaks was 3–19 (Mean = 6.6, SD = 4.0) years, and that of work after returning was 7 months to 8 years (Mean = 2.6 years, SD = 1.7 years). During the period of data collection, only two participants worked full-time, and 13 worked part-time. The areas of practice encompassed outpatient departments in hospitals (*n* = 8), hospital wards (*n* = 4), and long-term care facilities (*n* = 3) (see Table 2).

**Table 2:** The characteristics of the participants

ID	Sex	Age	Length of prior clinical experience (year)	Length of career break (year)	Length of clinical experience after returning	Employment status	Area of practice*	Number of beds in institutions	Reason for leaving job	Reason for returning to work
01	Female	30s	6	9	10 months	Part-time	Hospital (Outpatient)	146	Childbirth & child rearing	Encouraged by the return of a colleague
02	Female	30s	9	3	4 years & 5 months	Part-time	Hospital (Outpatient)	186	Childbirth & child rearing	Wanting to reconnect to society
03	Female	40s	4	5	2 years & 11 months	Part-time	Hospital (Outpatient)	186	Other	Encouraged by the return of a colleague
04	Female	30s	3	8	2 years & 3 months	Part-time	Hospital (Outpatient)	186	Childbirth & child rearing	Wanting to earn income
05	Female	30s	5	5	3 years & 3 months	Part-time	Hospital (Outpatient)	199	Childbirth & child rearing	Children growing up
06	Female	30s	9	3	2 years & 9 months	Part-time	Hospital (Ward)	199	Childbirth & child rearing	Not wanting to be a housewife
07	Female	30s	4	3	2 years & 8 months	Part-time	Hospital (Ward)	199	Childbirth & child rearing	Encouraged by family to return
08	Female	40s	5	19	2 years & 7 months	Part-time	Hospital (Outpatient)	199	Childbirth & child rearing	Encouraged by family to return
09	Female	30s	12	3	1 years & 2 months	Full-time	Hospital (Outpatient)	11	Care for parent	Wanting to utilize own clinical expertise
10	Female	30s	9	3	1 years & 11 months	Part-time	Long-term Care Facility	216	Childbirth & child rearing	Wanting to reconnect to society
11	Female	40s	10	10	8 years	Part-time	Hospital (Outpatient)	88	Childbirth & child rearing	Wanting to earn income
12	Female	30s	10	6	7 months	Full-time	Long-term Care Facility	46	Care for parent	Care for parent no longer required and children growing up
13	Female	40s	11	7	2 years & 9 months	Part-time	Long-term Care Facility	216	Marriage	Wanting to reconnect to society
14	Female	20s	4	4	1 years & 10 months	Part-time	Hospital (Ward)	190	Childbirth & child rearing	Children growing up
15	Female	70s	20	9	1 years & 9 months	Part-time	Hospital (Ward)	190	Care for parent	Care for parent no longer required

Note. * All the participants worked in private institutions not affiliated with any universities

The data analysis revealed five themes that facilitated the continuation of work for these participants. These themes include “Conditions and support that sustain work-life balance,” “A workplace that acknowledges my career, and encourages my growth as an experienced nurse,” “Pride in reconnecting with and contributing to society,” “Cultivating confidence through incremental professional development and future envisioning,” and “Enrichment of my own and family’s life.” The first two themes represent conditions that enabled the participants to continue their work. Thus, these conditions are referred to as “enablers”. The latter three themes describe factors that motivated the participants to pursue their professional careers. Thus, these factors are referred to as “motivators”.

### Theme 1: conditions and support that sustain work-life balance

The participants identified support systems at home, in the workplace, and within society as prerequisites for maintaining a work-life balance, essential for sustaining their employment. This theme encompasses crucial elements that allow nurses to balance their work and family responsibilities, such as work conditions that consider their family circumstances, and support from family and friends. The theme consists of three categories: “Work (i.e., hours and location) and childcare conditions that meet my preferences,” “A family-friendly work environment,” and “Instrumental and emotional support from family and friends.”

Most participants juggled work, household, and childcare responsibilities. Therefore, effectively managing childcare duties while fulfilling work roles became a priority in their lives. Access to childcare facilities was deemed a basic requirement for them to work, as well as conditions such as workplaces located close to their homes and offering flexible working hours to address child-related matters promptly.

*“When I was contemplating returning to work, one requirement was that I should be able to look after my two children, so it was important for me that all the conditions related to my children were in place, such as time restrictions and being able to go home immediately if something happens to them.” (ID 10)*.

The participants also emphasized the need for a family-friendly work environment, where colleagues and supervisors understood their family circumstances and provided support in balancing work and family duties.

*“When I returned to work, I wondered if I would be allowed to take a sudden leave if my child was ill. And they told me, ‘We take turns (taking a leave) so you can do it now, it’s fine,’ as well as ‘We can’t do it for you (take care of your child) but we can do the work in your place.’ Here at my current workplace, we can say such things to each other.” (ID 06)*.

Given that most participants were engaged in multiple tasks both at home and work, they experienced physical and mental fatigue and strain. However, they managed to overcome these challenges by receiving instrumental and emotional support from their families and friends. Examples of such assistance included husbands and children sharing household chores and friends providing emotional support during conflicts arising from the intersection of family and work responsibilities.

*“Regarding my husband, yes. When I started working, I was no longer a full-time housewife. But I’ve been working alongside him, and he’s been supporting me a lot, such as by taking the kids to school and picking them up after, things like that.” (ID 13)*.

Ensuring the effective management of household responsibilities, particularly childcare, was a fundamental prerequisite for the participants to continue their employment. Consequently, the provision of “Conditions and support that sustain work-life balance” acted as an enabler, facilitating their continued engagement in work by sustaining their personal lives.

### Theme 2: a workplace that acknowledges my career, and encourages my growth as an experienced nurse

The participants asserted that receiving support to cultivate their professional competencies within their work environment facilitated their transition through a process of reorientation. The participants were returners who had prior nursing experience and possessed a certain level of nursing competence required for professional practice. Initially apprehensive about their competence level, they desired recognition and appreciation for their previous experience and expertise from their supervisors and colleagues. They also expressed a preference for on-the-job refresher training that helped them regain necessary knowledge and skills. This training differed from that provided to newly graduated nurses. This theme represents the importance of receiving educational support to function as a nurse and opportunities for further growth, both of which facilitated the continuation of their work. The theme comprises three categories: “Supervisors and colleagues who appreciate and accept me,” “Support for myself as both a beginner and someone with experience,” and “Comprehensive manual and training.”

The participants emphasized the significance of being recognized and accepted by their colleagues and supervisors. The acknowledgment of their efforts by supervisors and the understanding of their hard work by colleagues served as encouragement to sustain their work. Furthermore, perceiving themselves as individuals who were relied upon by others and striving to meet those expectations facilitated their professional growth and their desire to contribute to the workplace.

*“One thing is that um, I also discussed this with the Head Nurse, regarding training, that maybe we should improve the training even more, and the Head Nurse feels the same way, and so, she said I can go ahead and think about a program or something. When I’m entrusted with making these kinds of decisions, the work becomes fulfilling.” (ID 09)*.

The participants also expressed the importance of receiving support from their colleagues as newcomers while appreciating their prior experience. The participants were often perceived as fully capable individuals and were assigned a workload equivalent to that of experienced nurses. However, the participants stressed the need for support from their colleagues during the initial phase of readjustment to their duties. Simultaneously, they sought appropriate levels of support while valuing their previous work experience and expertise. They felt reassured when their supervisors or colleagues offered support, recognizing them as both a beginner but also as someone with experience.

*“From the day after I started working, I had my own room, and on that day, someone from the day shift always made it a point to talk to me and support me, and it felt like fate. I thought if I were being supported this much, I should do the same, and well, everyone in the ward helped me understand the patients within the week, so much that I thought I already remember them. I felt that I should make an effort to do so, since they supported me so much.” (ID 06)*.

Additionally, they desired to receive training and manuals tailored to their skill set, enabling them to effectively perform their roles as staff members.

*“Although it was only 3 years, I did have a work gap, so I was thinking that my skills and knowledge might be obsolete and that I might have forgotten some things, but this hospital has a very detailed manual.” (ID 06)*.

Acceptance and support from both managers and colleagues, coupled with access to on-the-job training and manuals, emerged as crucial resources enabling participants to realign with their work responsibilities, especially in cases where they lacked up-to-date knowledge and skills. Additionally, feeling valued and trusted by colleagues played a pivotal role in bolstering their confidence, an essential attribute for navigating through challenging periods. Consequently, the provision of “A workplace that acknowledges my career, and encourages my growth as an experienced nurse” served as the pivotal enabler that sustained their professional life though continued commitment to their careers.

### Theme 3: pride in reconnecting with and contributing to society

The participants described working as nurses as giving them a sense of pride and of being valuable to society, which motivated them to continue their work. Prior to returning to work, the participants experienced social isolation due to their engagement in various household responsibilities. However, returning to the nursing profession allows the participants to reclaim their roles as active members of society and regain confidence in their contribution to society. The theme comprises three categories: “Desire to contribute as a nurse,” “Expansion of relationships resulting from stepping out of the home,” and “My children feeling proud of me for being an active nurse.”

The participants maintained a strong sense of pride in their profession and were motivated by the desire to contribute to society as nurses, utilizing their nursing qualifications. As the demand for nurses increased during the COVID-19 pandemic, their determination to support patients as nurses grew even stronger. They also expressed a desire to share their expertise with younger nurses and provide guidance to other inactive nurses who were considering returning to work.

*“Nurses are needed in situations like COVID-19, and I had gone through the trouble of getting my license, and all that.” (ID 03)*.

*“Well, I’d like to be in a position where people feel they can ask me and maybe find a bit of a solution. I work with the mindset that someone a bit older, like me, should take a role of listening to and giving advice to younger colleagues.” (ID 8)*.

Moreover, returning to work reaffirmed their sense of belonging to society not only as mothers but also as nurses. When they were solely focused on child-rearing, their social interactions were limited to those associated with their children. However, by returning to work and establishing their own place in the workplace, their social connections expanded beyond the confines of their homes. The opportunity to reconnect with broader society and experience personal freedom outside of their domestic responsibilities served as a motivation for the participants to continue their work.

*“It definitely connects me to society. Until now, my connections with society were through my child. I think I couldn’t have had that without my child, and now it feels like I have a separate community of my own. I feel like that.” (ID 08)*.

Furthermore, their pride in being nurses was reinforced by the admiration of their children, who proudly spoke of their mothers’ profession, especially during the challenging times of the pandemic. This alleviated any guilt associated with not having enough time to devote to their children and not fulfilling their maternal roles to the same extent as before. On the contrary, their professional engagement enhanced their self-esteem as proud mothers to their children.

*“When I think of these moments, it makes me really happy. Like those moments when I feel that my children have become interested in me (omitted). For example, when they say things like, ‘Nurses are really cool,’ or ‘My mom works in a hospital.’ They’ve even written about me in their diaries.” (ID 01)*.

Reclaiming a sense of pride and expanding their professional network through contributions to society represented profoundly fulfilling experiences for the participants. These experiences not only brought them joy in their work but also transcended the mere facilitation of work continuation. Consequently, “Pride in reconnecting with and contributing to society” operated as a potent motivator, driving their commitment to pursue their professional careers and advance, thus enriching their professional life.

### Theme 4: cultivating confidence through incremental professional development and future envisioning

The participants were motivated to continue their work by their passion for professional growth and self-actualization. The participants engaged in introspection regarding their journey from the moment they returned to work up until the present. Despite encountering challenging circumstances, they swiftly reacquired previously possessed skills and knowledge, thus restoring their self-assurance in the practice of nursing. This newfound confidence propelled them to envision their future career paths. The following three categories encompass this overarching theme: “Confidence arising from successfully surmounting challenges upon restarting,” “Realization that I have finally made my comeback as a nurse,” and “Personal aspirations for the future.”

According to the participants, they encountered arduous situations upon re-entering the workforce, as they were frequently required to perform tasks that exceeded their current skill sets. Irrespective of their absence from work, their colleagues often regarded them as seasoned nurses. Struggling to fulfill assigned responsibilities, they engaged in negotiations with colleagues and supervisors, asserting their capabilities and limitations. These challenging experiences facilitated the recovery and enhancement of the necessary skills and knowledge, bolstering their confidence, and motivating them to persevere in their work.

*“After returning to work, for about half a year, I struggled for a while before getting used to it again. It took me more than six months to understand why I was struggling. But when I got used to the working life, I was able to gain self-confidence.” (ID 04)*.

Through introspection and self-comparison between the time of restarting and the present, the participants recognized their continuous development as nursing professionals, observing their ability to provide a sufficient level of patient care.

*“In the sense that my intuition has returned, um, it was definitely the fact that before I started working, all I had was anxiety, but when I was actually able to perform my work by myself again, I think that was when I became confident.” (ID 10)*.

This developmental process stimulated their anticipation of future career prospects. Some participants expressed aspirations to acquire advanced qualifications and pursue managerial positions, thus making career advancement their future objective.

*“There was definitely something different about me, internally, before and after returning to work. It seems like I was lively, like I was going to set my goals, and that I was doing my best. There was a sense of certainty (omitted) and I was able to find what I wanted to do, too.” (ID 11)*.

The successful completion of the readjustment journey played a pivotal role in bolstering the participants’ confidence, and encouraged them to envisage future professional goals. The process of “Cultivating confidence through incremental professional development and future envisioning” emerged as a critical motivating factor (i.e., motivator), propelling the participants towards continued professional growth, and thereafter enriching their professional life.

### Theme 5. Enrichment of my own and my family’s life

The participants perceived added value when their own lives and their families were enriched by their work, which encouraged them to continue their jobs. The participants acknowledged the positive transformations in their physical and emotional well-being, as well as in the lives of their families, following their return to work. They perceived an overall improvement in their daily lives. This theme encompasses three categories: “A healthy mind and body attained by adding variety to life,” “Positive influence on family dynamics,” and “Income that enriches my life.”

The participants said that resuming employment contributed to a well-rounded lifestyle and positively impacted their physical and mental health. Specifically, those who were responsible for raising children noted that having time away from their children reduced feelings of irritability and enabled them to engage with their children in a more compassionate and nurturing manner upon returning home from work.

*“I feel like my day has become balanced. I do feel a little sad that I’m spending a lot more time away from my children (omitted). I make up for it when I see them, and I think I’ve become a little less irritable.” (ID 10)*.

Furthermore, having a job established a consistent rhythm to their lives and facilitated physical fitness, thus promoting a balanced existence. They also perceived the involvement of others in caring for their children as an opportunity for their children to interact with a broader network of individuals, fostering their growth and healthy development. Moreover, the up-to-date medical knowledge gained through their work served to safeguard the health of their families.

*“Because I want to know about cutting-edge technology. You know, if I quit this job, it will affect my life directly, because it’s a job that involves the body after all. I think it’s always gonna be useful (in my life).” (ID 13)*.

By earning their own income, they were able to provide economic security to their families, which was closely linked to their mental well-being.

*“Before I was reinstated, we were living on my husband’s salary alone. I felt bad about it, but now we have some financial leeway, so that definitely was a benefit for me.” (ID 11)*.

Resuming employment engendered an ‘Enrichment of my own and my family’s life,’ demonstrated by enhancements in physical and mental well-being, the wholesome development of children, and economic incentives. Consequently, this theme illustrates the enrichment of the participants’ personal lives as a result of having fulfilling professional lives, and emerged as an additional motivator.

## Discussion

This study explored factors contributing to the retention of nurses re-entering the workforce after a career break, resulting in the identification of five themes. The first two, “Conditions and support that sustain work-life balance” and “A workplace that acknowledges my career, and encourages my growth as an experienced nurse,” were identified as enablers, sustaining the participants’ continued engagement in work. The next three themes, “Pride in reconnecting with and contributing to society,” “Cultivating confidence through incremental professional development and future envisioning,” and “Enrichment of my own and family’s life,” served as motivators, propelling them toward a professional career.

**Fig. 1:**
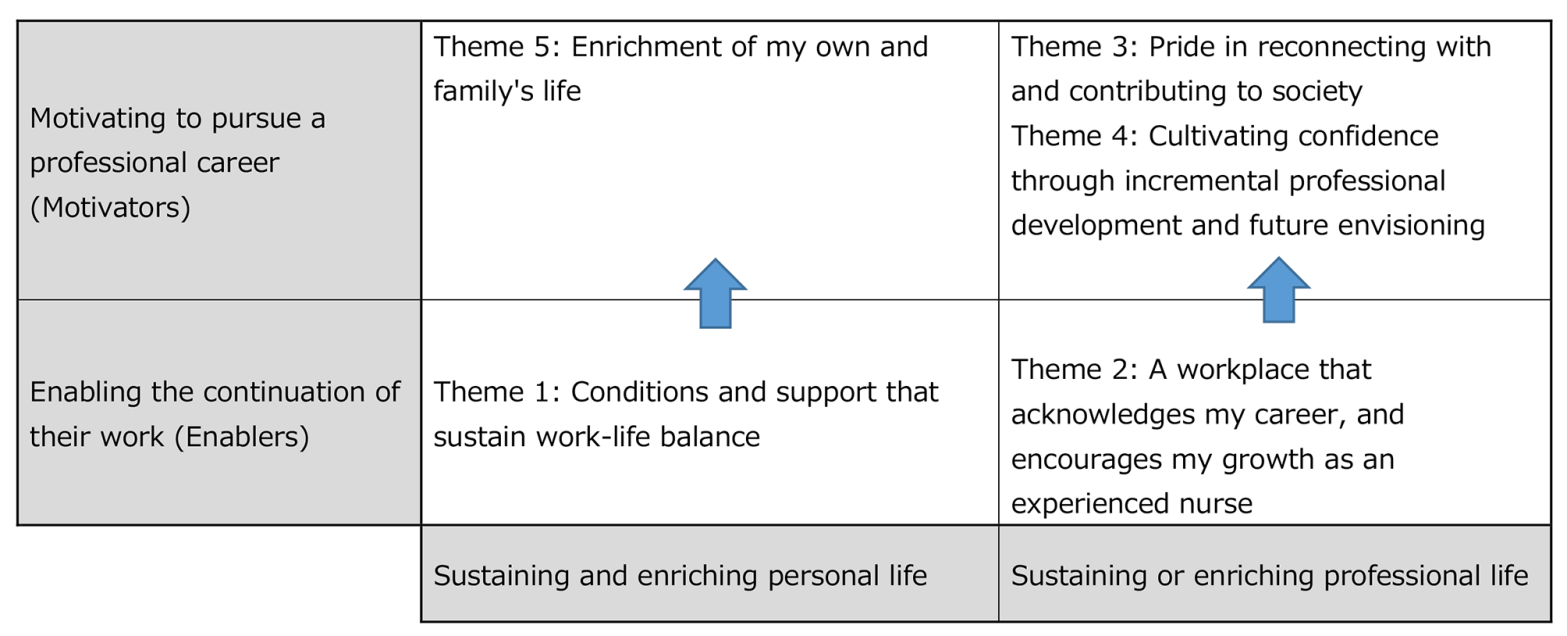
Framework for the sustainability of career for returners

The concept of enablers and motivators parallels Herzberg’s Two-Factor Theory of Motivation [43], where hygiene factors, including salary and work conditions, are essential but their absence leads to dissatisfaction, while motivation factors, like achievement and recognition, promote job satisfaction and enhanced performance [43]. Similarly, enablers such as family-friendly work conditions, peer support, and on-the-job training played pivotal roles in the participants’ job continuity, and their absence could result in dissatisfaction or job exit. Likewise, motivators such as pride and confidence yielded personal fulfillment, motivating participants to pursue their professional goals. However, distinctions arise. While the Two-Factor Theory focuses on work components, our study contends that healthcare institutions must address both professional and personal factors for nurse retention. This is critical, particularly for returning nurses, often with caregiving responsibilities, necessitating a balance between sustaining and enriching their professional and personal lives. Another distinction lies in the relationship between the enablers and motivators. According to the Two-Factor Theory, hygiene and motivation factors exist independently, while motivators do not exist without the presence of enablers. For example, without adequate support for nurses to achieve work-life balance, they are unable to enhance their own or their family’s quality of life. Similarly, lacking encouragement in professional development, nurses are unable to cultivate pride or confidence, or envision their future. These relationships are depicted in Fig. 1. The subsequent sections provide a detailed explanation of each of these factors.

The first theme, “Conditions and support that sustain work-life balance,” functions as an enabler that sustains nurses’ personal life. Nurses are prominent double-duty caregivers, tending to family and patients [44]. The majority of our participants had children, reflecting the fact that in Japan, 55–66% of nurses are parents [16, 45]. Therefore, balancing family and work is crucial, regardless of career breaks. Specifically, nurses who temporarily left their employment due to childcare responsibilities had various reasons such as the absence of available childcare support. Especially in Japan, women often prioritize their childcare responsibilities over work commitments, or may feel societal pressure to remain at home and care for their children [46]. These cultural practices and norms could potentially elucidate their career hiatus. Therefore, family-friendly working conditions (e.g., flexible hours, location, childcare support) are vital for returning and sustaining work. This finding is consistent with previous studies indicating that workplace flexibility, which helps alleviate childcare concerns, is crucial for enabling nurses to sustain their work [28, 30, 35, 36]. Furthermore, nurses who juggle dual caregiving roles often experience fatigue and stress [44]. Therefore, receiving instrumental and emotional support from their spouses is essential for maintaining a healthy work-life balance. In fact, recent studies have highlighted that support from their families enables nurses to effectively manage the demands of both their family and work spheres, facilitating their re-entry into professional practice [28, 35]. The successful sharing of household responsibilities and childcare is indispensable for returners who aspire to continue their professional work, particularly those with young children.

The second theme, “A workplace that acknowledges my career, and encourages my growth as an experienced nurse,” serves as an enabler that sustains the professional practice of returners. This finding is also in line with previous studies that have highlighted the significance of a supportive work environment in aiding individuals to manage their jobs and regain confidence [28, 35]. Although returners are often perceived as experienced nurses capable of functioning independently, the literature indicates that they encounter significant challenges in reacquiring their previous knowledge and skills, while also adapting to the rapidly advancing field of medical technology [21, 33, 35]. Reintegrating into the nursing workforce is arduous, and returners often experience anxiety and confidence issues [27, 31]. This was also evident among our participants. Consequently, receiving appropriate initial training and access to manuals are critical factors enabling returners to fulfill their duties and sustain their professional work [30]. On the other hand, the majority of the participants had achieved an expert nurse level, possessing more than five years of previous clinical experience [47], thus they desired recognition and acceptance of this. The need for acceptance and respect was also identified in previous studies on returning nurses [27, 30]. Appreciating their skills, efforts, and contributions while identifying areas for professional development represents the ideal “just-right preceptorship” for returners. Organizational support of this nature promotes work engagement [48], thus sustaining their professional practice.

While the existing literature commonly highlights the enablers necessary for nurses to return to work and continue their professional roles, previous studies have overlooked the motivating factors that drive them to work. Merely creating a sustainable environment for their return is insufficient. Internal drivers are essential to maintain their motivation to work, especially during challenging times. The following three themes describe the motivators that encourage nurses to pursue their professional careers, thus enriching their professional life.

“Pride in reconnecting with and contributing to society” stimulates nurses’ work motivation and enriches their professional lives. Previous studies have demonstrated that returning to work helps them regain self-esteem through their contribution to society, increasing pride as valuable society members [35, 36]. This study contributed new knowledge by highlighting how this sense of pride motivates returning nurses to pursue their professional careers. Nurses who had previously been inactive cited the desire to utilize their qualifications and contribute to the welfare of society as the main reason for returning to work [16]. They took pride in being nurses and were eager to apply their professional knowledge and skills, supported by their abundant clinical experience. This aligns with previous studies emphasizing their high levels of clinical and leadership skills [20, 28] and the enthusiasm exhibited by returners [30]. While initially struggling to adjust, their experience enables them to quickly adapt [33]. Once they regain competence, they contribute to healthcare and society by providing competent nursing care, educating colleagues, and serving as successful examples for potential returners. These experiences may instill a career calling characterized by self-actualization, personal fulfillment, and passion for their work [49], which promote job satisfaction [50] and engagement [51]. Returning to work also allows them to establish their societal position and expand their network, which is limited when solely fulfilling household responsibilities. According to the Self-Determination Theory [52], relating to others by engaging in employment outside the home not only alleviates isolation but also enhances their motivation. Additionally, contributing to society as valued members of the healthcare profession enhances their self-esteem [36] and allows them to cultivate a professional identity. If their children or significant others take pride in the nursing profession, their identification with nursing becomes stronger. During the COVID-19 pandemic, nurses were portrayed as heroes combating the crisis, which enhanced their professional identity and the pride their families had in them. Professional identity is known to enhance individual motivation to remain in the profession [53, 54]. Therefore, reconnecting with and making contributions to society enrich nurses’ professional lives.

“Cultivating confidence through incremental professional development and future envisioning” represents another motivator that enriches the professional lives of returners. Previous studies have shown the struggles and challenges that returning nurses faced in their journey towards reintegration, and in reaffirming their identity as nursing professionals [28, 31, 35]. When restarting their careers, returning nurses often experience anxiety due to changes within healthcare institutions, such as the introduction of new medical equipment and technology, shifts in insurance policies, increased demands for high-level physical assessment skills, and the expanded scope of responsibilities they now carry [55]. Nevertheless, the participants in this study successfully overcame numerous challenges and navigated the journey of reintegration. This experience of triumph and the acquisition of new knowledge and skills enabled them to regain the confidence they had in their previous career. Reflecting on their hard work and learning trajectory also instilled a sense of professional growth. Possessing confidence and a sense of self-worth has enhanced their self-efficacy, which, in turn, has promoted affective organizational commitment [56] and work engagement [57]. Furthermore, a successful reintegration fulfills their need for competence, thereby bolstering their motivation [52]. In addition. their learning achievements foster expectations for their future career goals. Having a clear goal enhances their professional development and further enriches their professional life. This study contributes new insights by demonstrating that perceiving their own professional development and embracing future goals motivates them to continue their work.

The final theme, “Enrichment of my own and family’s life,” highlights the reciprocity between personal and professional aspects for returners. Returning to work enables a balanced lifestyle, which improves mental and physical health and reduces strain and fatigue for double-duty caregivers. Employment also provides financial stability and enriches personal life, aligning with the previous findings [35]. Financial incentives are often cited as reasons for nurses to consider returning [23, 33]. While extrinsic, these incentives improve individuals’ quality of life, enriching their minds and energizing their work. Furthermore, work positively influences family dynamics, countering feelings of guilt at leaving children, often portrayed as a negative consequence of returning to work [31]. The participants in this study recognized the benefits, such as positive effects on their children’s healthy development, and how it led to an improved relationship with their children. Another study also observes a positive reciprocal relationship between work and family [35]. The theory of work-family enrichment asserts that " experiences in one role improve the quality of life in the other role” [58]. Work enriches personal life, while fulfillment in personal life motivates job continuation. Positive family experiences also enhance work performance [59]. Enrichment of personal life forms the foundation for individual professional life, and vice versa. This study reveals a new insight: returning to work can yield positive outcomes for nurses’ own lives and those of their families, particularly concerning child development.

### Implications for nursing management

The findings of this study suggest that in order to retain returners in the current nursing force, it is imperative to maximize both the enablers and motivators that contribute to the sustainability and enrichment of their personal and professional lives. In order to maximize the enablers, the establishment of a family-friendly environment is crucial. Nurse managers should strive to comprehend the personal and professional lifestyles that returners desire and should provide support accordingly. Furthermore, the formation of a mutual support group among returners can facilitate the exchange of experiences and encouragement, as well as make it possible to accommodate shift changes when family-related issues arise. The provision of adequate training is also of paramount importance. Unlike new graduate nurses, returning nurses possess diverse nursing skills and experience, necessitating a comprehensive evaluation by managers and colleagues to determine their competencies, while simultaneously providing them with the necessary knowledge and skills required for current clinical practice.

To enhance motivators, nursing managers should actively encourage returners to revive their professional pride and sense of fulfillment as nurses. One effective approach involves providing positive and constructive feedback on their contributions to the well-being of patients, thereby bolstering their pride. Additionally, managers need to assist returners in regaining their confidence and should support their progress toward achieving personal goals. Encouraging self-reflection on their clinical experiences can serve as a powerful means to help them realize the extent of their growth and subsequently enhance their confidence [31]. Assisting them in setting future professional goals represents another important strategy. Finally, managers should help returners recognize the positive changes that have occurred in their family dynamics as a result of their return to work. Engaging in discussions about personal life with managers or other returners may prove beneficial in this regard.

### Limitations

Efforts were made to enhance the transferability of the findings, by recruiting a heterogeneous sample of returning nurses, considering factors such as the duration of their career breaks, the length of clinical experience after returning, their employment status, and their area of practice. However, it cannot be assured that our sample is truly representative of Japanese returning nurses due to the relatively limited number of participants in this study. To enhance the transferability of the results, future studies should aim to replicate this research by encompassing diverse characteristics of returning nurses from various geographical locations. This approach would facilitate the aggregation of findings and the formulation of more robust programs designed to promote the retention of re-entering nurses.

## Conclusions

The nursing shortage is a persistent issue that is anticipated to worsen in the foreseeable future. The available solutions to alleviate this problem are limited, and a cost-effective approach involves incentivizing inactive nurses to rejoin the nursing workforce [60]. Returning nurses constitute a valuable asset for hospitals, as they possess a renewed professional commitment and can quickly regain nursing competence. Furthermore, their diverse experience in various clinical areas and organizations has the potential to introduce innovative clinical and managerial solutions within the current healthcare setting, thereby enhancing clinical outcomes and improving patient satisfaction. Therefore, it is imperative to implement multi-dimensional approaches aimed at retaining and harnessing the potential of these valuable human resources.

## Data Availability

The data are not publicly available because they contain information that could compromise the privacy of the research participants.
